# Rice Grain Quality and Consumer Preferences: A Case Study of Two Rural Towns in the Philippines

**DOI:** 10.1371/journal.pone.0150345

**Published:** 2016-03-16

**Authors:** Rosa Paula Cuevas, Valerien O. Pede, Justin McKinley, Orlee Velarde, Matty Demont

**Affiliations:** 1 Grain Quality and Nutrition Center, International Rice Research Institute, DAPO 7777 Metro Manila, Philippines; 2 Social Sciences Division, International Rice Research Institute, DAPO 7777 Metro Manila, Philippines; 3 Asian Development Bank, 6 ADB Avenue, Mandaluyong City 1550, Metro Manila, Philippines; Wuhan University, CHINA

## Abstract

Hedonic pricing analysis is conducted to determine the implicit values of various attributes in the market value of a good. In this study, hedonic pricing analysis was applied to measure the contribution of grain quality search and experience attributes to the price of rice in two rural towns in the Philippines. Rice samples from respondents underwent quantitative routine assessments of grain quality. In particular, gelatinization temperature and chalkiness, two parameters that are normally assessed through visual scores, were evaluated by purely quantitative means (differential scanning calorimetry and by digital image analysis). Results indicate that rice consumed by respondents had mainly similar physical and chemical grain quality attributes. The respondents’ revealed preferences were typical of what has been previously reported for Filipino rice consumers. Hedonic regression analyses showed that grain quality characteristics that affected price varied by income class. Some of the traits or socioeconomic factors that affected price were percent broken grains, gel consistency, and household per capita rice consumption. There is an income effect on rice price and the characteristics that affect price vary between income classes.

## Introduction

Rice *(Oryza sativa* L.*)* is one of the most important food crops as it is consumed by more than half of the world’s population [1]. Despite its importance, the international rice market is considered a “thin” market; it is highly segmented because rice consumers have very specific preferences [2]. The definition of “premium-quality” rice is largely dependent on the socioeconomic context of consumers, with data suggesting that even lower income classes are increasingly conscious of food quality [3–6].

Rice quality is judged based on attributes, which could be classified several ways. Product characteristics could either be *intrinsic*, such as taste, texture, or color; or *extrinsic* to the product, such as packaging, brand, or label. Another attribute classification distinguishes between *search*, *experience*, and *credence* attributes. Search attributes are available for product evaluation before purchase, such as price, appearance, brand, and packaging. Experience attributes can be evaluated only upon product experience, thus after purchase or product use—examples are taste, texture, ease of cooking, and swelling capacity. Credence attributes are attributes that consumers cannot evaluate or verify themselves. Instead, they rely on people or institutions, such as government controls or industry claims. Attributes relating to production, processing, and product contents are typical examples of the credence-type attributes [7]. In this paper, we will focus on *intrinsic search* and *experience* attributes, such as visual and physicochemical grain properties. It is argued that measuring such properties objectively is difficult [8] but relatively high-throughput routine methods have been developed to conduct measurements of a number of rice quality parameters.

Visual characteristics of rice grains are important *search* attributes that affect consumers’ purchasing decisions and hence are used as some of the first selection criteria in varietal improvement programs [9–11]. Grain size is mainly based on the length. On the other hand, grain shape is based on length-to-width ratio [10]. The classification of rice samples based on size and shape is not standardized across different countries and different markets [12–14]. The routine classification system used by the International Rice Research Institute (IRRI) breeding programs is as follows: short (≤ 5.50 mm), medium/intermediate (5.51–6.60 mm), long (6.61–7.50 mm), and very long (> 7.50 mm). The grain shapes of rice, likewise, can be described based on the routine value ranges used in IRRI: bold (≤ 2.0), medium (2.1–3.0), and slender (> 3.0) [14]. Chalky areas in rice grains—those opaque white parts of the grain—are deemed, generally, to represent poor quality in many rice market segments and thus these grains fetch lower market prices [15]. Grains are classified based on the proportion of the grain that is chalky: none (0%), small (< 10%), medium (10–20%), and large (> 20%) [14,16]. Traditionally, rice grain dimensions were measured using photographic enlargers and transparent rulers [9] while visual scoring by an experienced technician was conducted to determine chalkiness in rice grains [10]. Using manual ways of measuring grain dimensions is laborious and time-consuming while visual assessment of chalk has some degree of subjectivity and does not indicate where the chalky portion is in the grain [17].

*Experience* attributes, such as cooking and organoleptic properties of rice, affect a consumer’s repeat purchasing behavior. Three parameters deemed most important in gauging the cooking and eating quality of a rice variety are: apparent amylose content (AAC), gel consistency (GC), and gelatinization temperature (GT). As AAC increases, cooked rice grains tend to be increasingly harder [18]. Colorimetry with iodine [19–21] remains the method of choice for measuring AAC despite its limitations [18,22–24] and the development of new methodologies summarized by [25]. Based on AAC, rice can be grouped into five arbitrarily set classes: waxy (0–2%), very low (3–9%), low (10–19%), intermediate (20–25%), and high (> 25%) [26] although a more recent study suggests that these AAC classes can further be subdivided [27]. There are cases in which rice materials of the same AAC class are very distinct in hardness. In these cases, GC is used as a complementary test for degree of hardness upon retrogradation. The methods for measuring GC, or the hardness of rice upon cooling after being cooked, are still in routine use today mainly for rice breeding programs focused on intermediate- and high-AAC materials [27,28] while a method has been developed for glutinous rice [29]. Rice can be classified into three groups based on GC: hard and very flaky (≤ 40 mm), medium and flaky (41–60 mm), and soft (> 61 mm) [10]. On the other hand, GT is associated with the cooking time of rice [30,31]. Rice can be classified based on GT: low (< 70°C), intermediate (70–74°C), and high (> 74°C) [32]. The alkali spreading test [33] is a high-throughput assay for GT but this entails some subjectivity since the scores are based on perceptions of the analyst. Scores indicate GT classes, not direct information regarding the GT of the rice sample.

Throughout the world, consumer preferences are far from homogeneous. Various market segments can be distinguished between continents, regions, countries, and even between socio-economic groups [7,34,35]. Grain quality experts in 23 countries have identified the top three popular rice varieties in their countries and, for some countries, at various sub-country levels; the most commonly assessed cooking and eating properties of these varieties have been reported [27]. Consumers may not be able to articulate the reasons behind their preferences or describe what they like or dislike in food items but they show appreciation or the value they attach to food in other ways [36] such as a willingness to pay higher prices for rice with certain quality attributes. Price differences between rice samples of different quality classes indicate that grain quality attributes must be contributing to the price of rice.

Determining the implicit contribution of the various grain quality attributes to the market price of rice varieties can be done through hedonic pricing analysis [37]. Hedonic pricing regressions have been quite popular in the economics literature, being applied to various food commodities such as wine [38,39], tea [40], apples [41], and breakfast cereals [42]. In all these commodities, the products could be grouped into quality classes or varieties [43]. The hedonic pricing model has also been applied to study the effects of extrinsic and intrinsic quality attributes of rice to market prices [44–47]; with the results suggesting that varietal improvement programs should not be limited only to yield-enhancing traits. Furthermore, dissemination and adoption of new varieties need to be supported. A recent study in Central Luzon found that out of the 200 modern rice varieties released, fewer than 10 varieties are being used by farmers in the Central Luzon area [48].

Previous hedonic studies involving intrinsic rice quality parameters [44,46,47] have two main limitations. First, they did not investigate how homogeneity in some physical characteristics influences rice prices. Homogeneity in physical characteristics—such as length and width—of the rice sample being purchased may play a major role in consumers’ willingness-to-pay for rice. Rice varieties are often mixed at various stages of harvest and post-harvest activities (i.e. harvesting, threshing, drying, and milling), which results in heterogeneous grain quality. Second, rice quality data obtained in these studies, specifically degree of chalkiness and GT, were measured through semi-quantitative means: scores were provided based on experienced technicians’ evaluations. Techniques that provide quantitative data now exist and can potentially improve hedonic pricing models. Machine vision technology, such as digital imaging systems, is available for monitoring quantifiable attributes of post-harvest quality of plant and animal products such as size, shape, and degree of chalkiness, as in the case of rice [17,49–51]. Differential scanning calorimetry (DSC) is an alternative method (to the alkali spreading test) for characterizing GT; it monitors thermal transitions and provides the temperature range at which the crystalline starch structures irreversibly melt in the presence of plasticizing water [52,53]. Paste viscosity, measured using a Rapid Visco-Analyzer (RVA), is another indicator of cooking and eating quality in rice [54,55]. Different parts of the viscosity curve have been associated with GT, with AAC, and with texture [55–57].

Previous literature on consumer preferences in the Philippines has mainly focused on big urban consumption zones [44,47,58]. Since the Philippines is a net importer, consumer preferences in urban areas close to the port tend to be dominated by imported rice characteristics, which are not necessarily satisfied by domestic supply. In order to focus on consumer preferences for the characteristics of rice varieties which are currently produced domestically, we need to move away from these highly urbanized zones. Since we are also not interested in preferences of consumers who are producers themselves, we need to look for concentrated consumption zones close to production zones, i.e. rural towns. Therefore, in this study, we determine how the implicit market value of intrinsic search and experience quality attributes of rice contribute to the total market price of rice in two rural towns in the Philippines: Famy (14.4333°N, 121.4500°E) and Sta Maria (14.4700°N, 121.4261°E). In doing so, the contributions of this study are: (1) to determine consumer preferences in rural areas, which have largely been de-prioritized in consumer preference studies; (2) to apply quantitative methodologies in measuring GT and chalkiness; and (3) to identify the household willingness-to-pay for various characteristics of rice by income groups.

## Materials and Methods

### Ethics statement

There is no Institutional Review Board at IRRI at this time. However, this work has been reviewed and approved by the division head of the Social Sciences Division through an internal review process. All participants in this study gave informed oral consent prior to the survey interview and had the option to terminate the interview at any point. No minors were directly interviewed during this study. All datasets collected by the Social Sciences Division of IRRI are ultimately uploaded and made available for public use. However, it is our policy to first make datasets anonymous prior to uploading. This is done by removing all identifying information within the dataset, including: name, email, telephone number, street address, and gps coordinates. These measures are done with the approval of the Chief Information Officer of the International Rice Research Institute.

### Survey and sample collection

Rice consumer respondents (n = 128) were selected in the adjacent towns of Famy and Sta Maria, two northernmost towns in Laguna province in the Philippines, situated at 93 and 86 km from the capital city, Manila, respectively. These towns are both rural areas. To select the respondents, high-resolution imagery from Google Earth and a global positioning system (GPS) were used. The target population was delimited in the town proper because houses are more closely situated there. Households 20 meters apart were marked based on GPS coordinates. Then, 100 GPS coordinates were randomly selected. In cases when the GPS coordinates pointed to vacant lots or roads, respondents were obtained from the house nearest the GPS point. All respondents gave oral informed consent to be surveyed.

Respondents were interviewed to determine their socioeconomic profiles (Table 1). A sample of uncooked milled rice (300 g) consumed by these respondents was then collected in exchange for 1 kg of premium milled rice. At the time of collection, respondents also reported the price paid per kilogram of rice. The milled rice samples from the respondents were sent to the Grain Quality and Nutrition Center of the IRRI for physicochemical analyses. For the statistical analysis presented in this paper, households were classified based on Philippine National Statistics Office (NSO) income classes [59] but with a slight modification by merging the three lowest NSO-reported income classes into a single category as the low household income class used for this study. For the final analysis, only three income classes remain: low household income (< 2,431.91 USD per annum), medium household income (between 2,431.91 and 6,079.77 USD per annum), and high household income (>6,079.77 USD per annum). The exchange rate at the time of the study was 1 USD = 41.12 PHP

**Table 1:** Characteristics of respondents by income group and by location (Famy and Sta Maria, Laguna, Philippines).

	Income Group			Location		
	Low	Middle	High	Famy	Sta Maria	Combined
Household size	4 (1)	5 (2)	5 (2)	5 (2)	5 (2)	5 (2)
Annual household income (USD)	1,512.79 (657.56)	3,923.47 (991.44)	10,305.86 (4,836.09)	5,366.85 (4,335.58)	4,910.46 (4,829.18)	5,145.79 (4,568.85)
Age of rice purchaser (yrs)	43 (15)	46 (15)	43 (13)	43 (15)	45 (14)	44 (15)
Educ. of rice purchaser (yrs)	9 (3)	10 (3)	11 (3)	10 (3)	10 (3)	10 (3)
Rice consumption per capita (kg)	211 (91)	214 (107)	200 (86)	230 (102)	186 (83)	209 (95)
Rice price (USD/kg)	0.78 (0.05)	0.80 (0.05)	0.83 (0.07)	0.80 (0.05)	0.80 (0.07)	0.80 (0.05)
Sample Size	41	47	40	66	62	128

Note: Figures presented here are means and standard deviations (in parentheses). The conversion rate at the time of the study (September to December 2012) was 1 USD = 41.12 PHP.

### Grain quality analyses

Milled grains underwent assessment of physical traits (grain dimensions, proportion of head rice in milled rice, and chalkiness) and then an test portion of each sample was ground into fine flour (100-mesh) using a Udy Cyclone Sample Mill (model 3010–30, Fort Collins, CO). Reverse osmosis (RO) water and reagent-grade chemicals were used for the chemical analyses.

Physical traits (length, width, and degree of chalkiness) of the milled rice grains were determined using the Cervitec^™^ 1625 Grain Inspector (FOSS, Denmark). Grain shape was calculated based on the length-to-width ratio of the grains. The proportion of head rice (%) in the milled rice was determined by measuring the amount of grains that are 75% intact after a test portion (100 g) of milled rice was sorted using a shaking sieve; the rest are broken grains (%). The measurement of AAC was conducted following the Routine Method of ISO 6647 [60], and calculated based on a standard curve generated using the iodine-binding capacities of a set of standard rice varieties. The AACs of these standards were determined as described in the Reference Method of ISO 6647 [61]. Absorbance was measured at 620 nm using a San^++^ Automated Wet Chemistry Analyzer (Skalar Analytical B.V., Breda, The Netherlands) equipped with an SA1100 sampler. Data were collected and analyzed using the Skalar FlowAccess^™^ V3 software. Gel consistency was determined according to a previously published protocol [28]. Gelatinization temperature was measured by DSC (Q100, TA Instruments, New Castle, DE, USA). Flour (4 mg) and RO water (8 μL) were placed in an aluminum pan, which was then sealed hermetically. An empty hermetically sealed pan served as the reference. The temperature was raised from 35°C to 120°C at 10°C min^-1^. Thermal transitions were recorded and analyzed using the TA Universal Analysis 2000 software. The peak of each resulting endotherm was reported as the GT. Viscosity curves for the rice samples were generated using the RVA following the profile detailed in the AACC Method 61–02 [62]. Several points in the viscosity curves were recorded: peak (PV), trough (TV), and final (FV) viscosities; derived values from these points were calculated: breakdown (BD, the difference between PV and TV), lift-off (LO, the difference between FV and TV), and setback (SB, the difference between FV and PV) [63]. The time of PV (peak time) and pasting temperature were also obtained.

### Statistical analysis

#### Across income groups

One-way analysis of variance (ANOVA) was conducted for hypothesis testing for grain quality parameters that passed the assumptions of ANOVA (average length, variability in length, PV, TV, FV, and LO). For the other parameters, hypotheses were tested using Kruskal-Wallis rank sum test and means were compared using the Mann-Whitney-U post-hoc test with Bonferroni correction.

#### Between towns

The Z-test was used to compare samples from the two towns in the quality parameters whose data were normally distributed. For parameters whose data were not normally distributed, the Mann-Whitney (Wilcoxon rank sum) test was used.

These analyses were conducted using R (version 3.2.0, released 2015).

### Establishing the hedonic price model

Hedonic pricing regressions are based on Lancaster’s “characteristics theory of value,” which states that any good can be described in terms of its attributes or characteristics [64]. The price consumers are willing to pay for a good at a given time is therefore assumed to depend on the attributes of the good or commodity.

When buying rice, consumers face a choice of several search attributes, some are visible and others are not, but those attributes are embedded in the product. Each of these attributes contributes to the final price paid on the market. The socioeconomic status of consumers and their preferences will determine which products they will buy among a given set of quality-differentiated rice types available in the market and hence which prices they are willing to pay for those products. The rice types purchased and prices paid by consumers can be interpreted as their revealed preferences and willingness-to-pay (WTP) [65]. Thus, besides the physical and chemical characteristics of rice, we consider that consumers’ socioeconomic status also determines the type of rice they choose in the market and the price they are willing to pay. We therefore specify our hedonic pricing model as follows:

$$P_{i}=\beta x_{i}+\rho z_{i}+\rho k_{i}+\eta D+\varepsilon_{i}$$ (1)

where, *P* represents the price paid for rice by consumer *i*, **x** is a vector of physical attributes characterizing rice purchased by consumer *i*, **z** is a vector of chemical characteristics embedded in rice purchased by consumer *i*, **k** is a vector of socioeconomic characteristics describing consumer *i*, *D* is a location dummy assumed to capture region-specific factors, and ε is the error term of the model.

The hedonic model expressed in Eq 1 is estimated in log-log functional form using Ordinary Least Squares (OLS). The model was first estimated as a pooled sample, then per income group, and with income interaction terms on physical and chemical rice characteristics and one socio-economic factor (per capita rice consumption). The physical characteristics considered in the model are: the proportion of broken rice in the sample and chalkiness. The chemical characteristics are: GC, AAC, and GT. The consumer characteristics are: rice consumption per capita, age, gender, and education of the rice purchaser.

Traditionally, the estimated coefficients from the hedonic regression are interpreted as consumers’ WTP for a given attribute of the good or commodity. A positive sign indicates that consumers are willing to pay a price premium for the attribute, while a negative sign reveals that consumers discount the attribute.

Studies on consumer preferences typically use consumer and expert surveys or interviews [27,66], quality evaluations of samples coming from national programs [58,67], or consumer product preference and acceptability tests using specific sets of rice samples [68]. Such approaches, although effective in determining stated consumer preferences and the characteristics of those rice varieties, do not indicate buyers’ WTP based on quality attributes. To reveal realistic contributions of grain quality to market prices in rice that people actually buy, it is best to base hedonic regression models on information about samples obtained from consumers.

This method of obtaining sample from surveyed households was previously employed by Abansi et al. [44]. In that study, rice samples obtained from surveyed households were analyzed for physicochemical characteristics to compare consumer preferences between urban and rural consumers [44]. Additionally, consumer preferences were investigated across different income strata.

## Results and Discussion

### Socioeconomic characteristics of the respondents

Among the 128 respondents surveyed (Table 1) 66 came from Famy and 62 from Sta Maria. The 128 respondents were also grouped by income classes: 41 were classified as low-income, 47 as middle-income, and 40 as high-income.

The respondents in Famy and in Sta Maria had similar socioeconomic characteristics and, on average, bought rice with the same price (0.80 USD/kg, Table 1). The majority (80%) of the rice purchasers who participated in the survey have studied for 8–12 years, with age averaging in the 40s across the three income classes (Table 1). Household sizes of respondents, on average, were four household members for the low-income class and five members in both middle- and high-income classes, and in both towns. The annual rice consumption of the respondents in these households was mainly 100–250 kg per capita. The averages of per capita consumption of rice by location and by income class (Table 1) were higher than the per capita consumption determined in the 1990s for rural consumer groups [69] and the national average in 2008–2009 [70]. Most of the respondents earned less than 4,863.81 USD annually, with the number of respondents decreasing with an increase in income.

### Physical characteristics of raw grains of the rice samples

Based on the IRRI classification system for grain size and shape [14], the respondents across the different income groups in the towns had a revealed preference for rice with long and slender grain shape (Tables 2 and 3).

**Table 2:** Physical, cooking, and eating quality indicators of rice samples obtained from respondents by income class.

	Income Class				
	Low	Middle	High	p-value^c^	Combined
Grain length (mm)	6.67 (0.08)	6.67 (0.09)	6.69 (0.08)	0.35^A^	6.68 (0.08)
CV in length (%)	4.76 (0.47)	4.66 (0.36)	4.86 (0.41)	0.09^A^	4.76 (0.41)
Width (mm)	2.08 (0.06)	2.10 (0.06)	2.10 (0.05)	0.30^B^	2.09 (0.06)
CV in width (%)	7.94 (0.82)	7.77 (0.02)	7.87 (0.78)	0.52^B^	7.85 (0.75)
Ratio of length/width	3.20 (0.11)	3.18 (0.11)	3.19 (0.08)	0.43^B^	3.19 (0.10)
Chalkiness (%)	17.00 (8.00)	19.00 (8.00)	17.00 (7.00)	0.13^B^	18.00 (7.00)
Head rice (%)	56.75 (8.85)	55.56 (11.13)	58.49 (14.41)	0.70^B^	56.86 (11.61)
AAC (%)	24.01 (1.98)	23.54 (2.32)	24.12 (1.46)	0.78^B^	23.87 (1.98)
GT (°C)	77.37 (1.07)	77.04 (1.70)	76.76 (2.41)	0.32^B^	77.06 (1.80)
GC (mm)	50.90 (12.10)	54.20 (13.50)	51.60 (14.80)	0.49^B^	52.30 (13.50)
PV (cP)	2924.00 (317.00)	2922.00 (290.00)	2809.00 (325.00)	0.16^A^	2887.00 (312.00)
TV (cP)	1638.00 (256.00)	1637.00 (229.00)	1617.00 (287.00)	0.92^A^	1631.00 (255.00)
BD (cP)	1287.00 (228.00)	1285.00 (335.00)	1192.00 (222.00)	0.10^B^	1257.00 (272.00)
FV (cP)	3900.00 (517.00)	3850.00 (480.00)	3908.00 (613.00)	0.86^A^	3884.00 (533.00)
SB (cP)	975.00 (422.00)	928.00 (578.00)	1099.00 (497.00)	0.49^B^	997.00 (508.00)
Peak time (min)	5.56^a^ (0.09)	5.5^a^^b^ (0.09)	5.52^b^ (0.09)	0.03^B^	5.54 (0.09)
Pasting temperature (°C)	75.46 (0.96)	75.10 (1.64)	75.38 (1.31)	0.58^B^	75.30 (1.35)
LO (cP)	2262.00 (298.00)	2213.00 (303.00)	2291.00 (364.00)	0.52^A^	2253.00 (321.00)

^a^ Figures presented here are means and standard deviations (in parentheses). For peak time, a different lowercase letter beside each mean indicates that the means are significantly different (α = 0.05).

^b^ The conversion rate at the time of the study (November 2012) was 1 USD = 41.12 PHP.

^c^ The letter beside the p-value indicates the test statistic used: (A) ANOVA (F-statistic), (B) Kruskal-Wallis rank sum test (χ^2^).

**Table 3:** Comparison of physical, cooking, and eating quality indicators of rice samples obtained from respondents by location (Famy and Sta Maria, Laguna, Philippines) using the Z-test.

	Location		
	Famy	Sta Maria	Z-value
Grain length (mm)	6.66 (0.08)	6.70 (0.08)	-2.55
CV in length (%)	4.72 (0.43)	4.80 (0.41)	-1.05
PV (cP)	2,839.00 (307.00)	2,939.00 (311.00)	-1.82
TV (cP)	1,613.00 (248.00)	1,649.00 (263.00)	-0.79
FV (cP)	3,872.00 (530.00)	3,897.00 (540.00)	-0.26

Note: Values presented are means and standard deviations (in parentheses).

At p < 0.05, attributes with Z < -1.96 or Z > 1.96 are significantly different between the locations.

Results indicated that the widths and the shapes of rice samples obtained from respondents in Famy and in Sta Maria were not significantly different across income classes (Table 2) and between towns (Table 4). The lengths of the rice grains were not significantly different across income classes (Table 2) but there was a small but significant difference in the length between the grains consumed in the two towns: Famy respondents purchased, on average, slightly shorter grains than Sta Maria respondents (Table 3), but the difference was not large enough to put the grains into different quality classes. The variability in length and in width of rice grains across the different income classes and between towns were not significantly different as well (Tables 2–4). The data indicated a preference for long and slender grains. This similarity may be associated with the proximity of the two towns. It is possible that the markets in these towns have the same set of rice suppliers, hence leading to the same grains being sold. It is also possible that this preference for long and slender grain is stable due to similarities to grain dimensions of IR64 in the late 1980s [71], a benchmark of rice grain quality in the Philippines for millers, traders, and consumers [72]; and to recently reported expert opinions on grain dimensions of highly preferred Filipino rice varieties [27].

**Table 4:** Comparison of physical, cooking, and eating quality indicators of rice samples obtained from respondents by location (Famy and Sta Maria, Laguna, Philippines) using the Mann-Whitney (Wilcoxon rank sum) test.

	Location		
	Famy	Sta Maria	p-value^a^
Width (mm)	2.09	2.09	0.98
CV in width (%)	7.79	7.92	0.26
Ratio of length/width	3.19	3.2	0.35
Chalkiness (%)	18	18	0.75
Head rice (%)	56.71	57.01	0.99
AAC (%)	23.66	24.1	0.06
GT (°C)	77.09	77.04	0.39
GC (mm)	53.65	51.06	0.21
BD (cP)	1226	1290	0.24
SB (cP)	1033	958	0.9
LO (cP)	2,259	2,248	0.55
Peak time (min)	5.52	5.56	0
Pasting temp (°C)	75.27	75.38	0.31

Note: Values presented are means.

^a^ For comparison between locations, attributes with p < 0.05 are significantly different.

Chalky areas in rice grains are caused by loose packing or incomplete filling of starch granules [15,31,73]. It effectively weakens the grain [31,74], leading to elevated incidence of breakage during the milling process and to reduced head rice yield [75]. Across the income classes and between towns, the degree of chalkiness was not significantly different, with the grains having medium chalkiness, on average (Tables 2 and 4). These findings indicate that the respondents in this study have similar preferences in terms of chalkiness in grains. Perhaps, the respondents did not mind having opaque spots on the raw rice grains as long as grains are not broken. After all, chalkiness does not directly affect the cooking and eating experience of rice [16].

There were non-significant differences in the proportions of head rice across the different income groups (Table 2) and between the two towns (Table 4), with respondents having submitted samples with 57% head rice, on average. Based on the proportion of head rice in the respondents’ samples, it appears that the respondents were willing to pay for milled rice that fell below premium standards set by the Philippines’ National Food Authority (NFA) [76]; perhaps, premium grade milled rice was not available in the markets surveyed in this study. However, the data reported here (Tables 2 and 4) indicate improvement from previously reported rice mill yields in the Philippines [77], suggesting that post-harvest processing conditions and processing facilities have improved. The motivation of breeders and post-harvest practitioners to improve head rice recovery could stem from consumers’ possible association between good taste and the wholeness of the rice grain. It is possible that the respondents’ choices of rice were constrained by their purchasing power as head rice has been reported to be correlated with market rice prices [78].

### Cooking and eating properties of the rice samples

Amylose is one of two starch polymers in rice. Amylose content is believed to be one of the best single indicators of the texture, particularly of the hardness, of rice samples [24]; hence, it plays a critical role in selection decisions in rice breeding programs [23]. In this study, there were no significant differences in AACs in rice consumed across the different income classes (Table 2) and between towns (Table 4), with the respondents consuming rice with intermediate AAC, on average. These findings agree with previously reported Filipino consumer preferences [27,34].

On the other hand, the rice samples from the respondents were, on average, of the medium GC class, in agreement with characteristics of popular Philippine rice varieties reported by Calingacion et al. [27]. The respondents’ textural preferences, according to GC values, were similar across income classes (Table 2) and between towns (Table 4). Amylose has been implicated in affecting GC [53], which predicts texture of cooked rice; however, rice texture is also reportedly influenced by proteins and lipids [63,79–82].

Amylopectin is the other polymer of starch in rice grains. During gelatinization, the crystalline lamellae of amylopectin melt; the temperature range at which this happens—referred to as GT—depends on the distributions of chain-lengths within the amylopectin semi-crystalline cluster [30,83–84]. Respondents across income classes (Table 2) and between towns (Table 4) similarly preferred rice with high GT. This result contrasts reports that indicated that the preferred GT class in the Philippines is low to intermediate [27,85]. However, DSC could give a different value from what is obtained from alkali-based methods [30], which have been used for GT characterization in the contrasting studies. Juliano et al. had used the alkali turbidimetric assay [85] while Calingacion et al. obtained ASV values from the experts who participated in the survey, only conducting DSC for samples of unknown GT (the authors did not specify which samples were subjected to DSC or had reported ASV data) [27].

The rice submitted by the respondents had statistically similar viscosity values across income groups (Table 2) and between towns (Tables 3 and 4). However, there was a small but significant difference in the amount of time the rice samples needed to reach PV among the different income groups. The low-income group had rice samples with slightly longer peak times than samples obtained from the high-income group (Table 2). The difference in peak times indicates slightly different swelling behaviors between the rice from the low-income group and the high-income group; its impact during cooking, however, might be too small to be distinguished as GT is similar across income classes (Table 2).

### The hedonic price model

**Fig 1:**
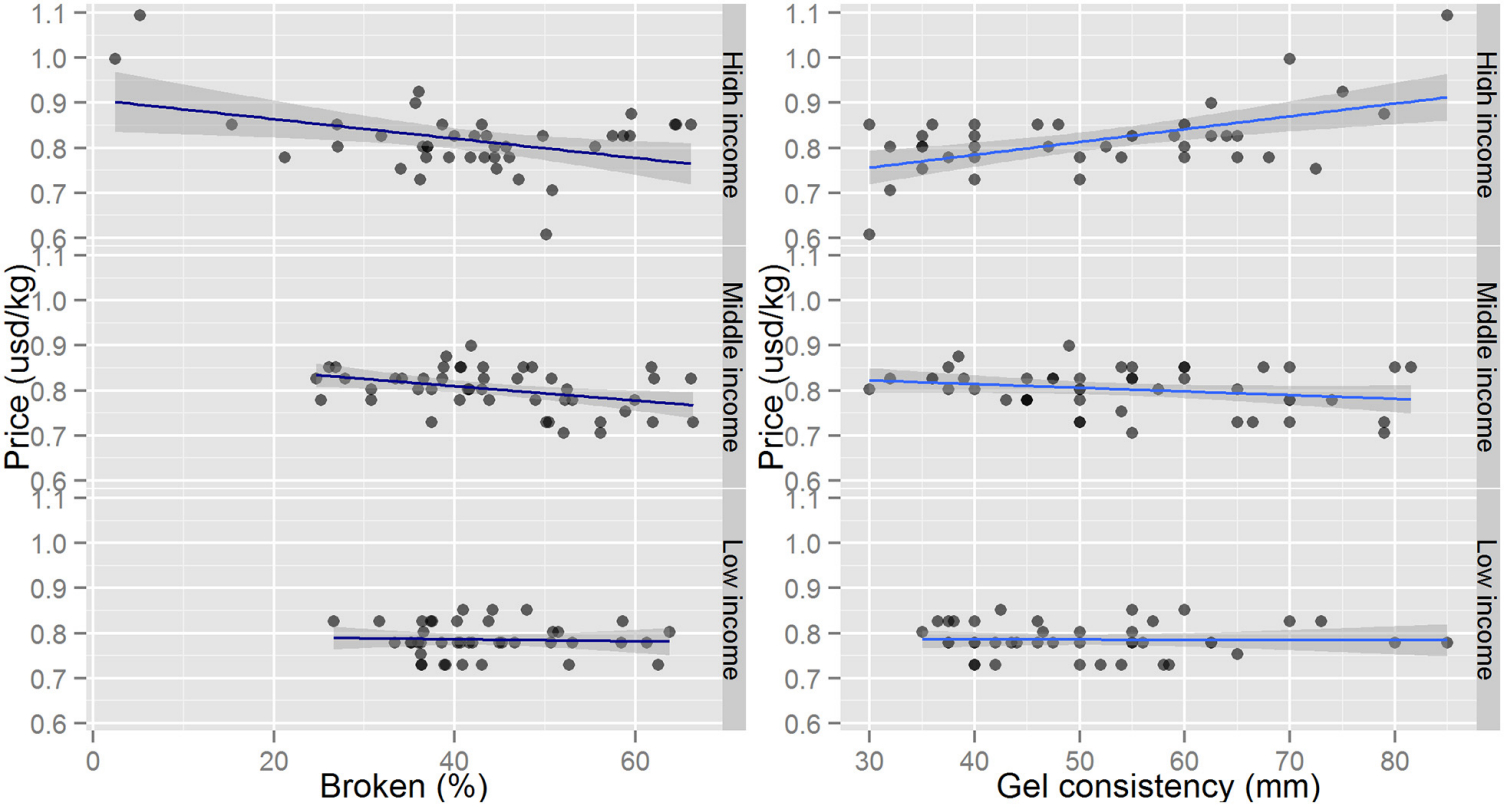
Results of correlation of price with percent broken and gel consistency by income class.

This study uses a log-log functional form in all preliminary models (Table 5) as well as the final model (Table 6). One advantage of this functional form is that estimated coefficients can be interpreted as elasticities. The original hedonic model that was investigated can be found in column (1) of Table 5. In this model, per capita income was found to be significant; different income levels are likely to have different hedonic price models. Therefore, we split the same regression in three income classes (columns (2) to (4)) and observe that slopes of explanatory variables such as percent broken and GC indeed differ across income strata (Fig 1). Unfortunately, this method also has the disadvantage that sample sizes for income groups are smaller and degrees of freedom become limiting due to the inclusion of several explanatory variables on rice grain quality as well as socio-economic factors. Because of this limitation, income classes are interacted with grain quality characteristics and one socio-economic factor in Table 6. Other socio-economic factors were found to have similar effects among income classes. Hence, we were able to exploit the full, pooled sample and significantly increase the explanatory power of the original model (column (1) in Table 5) from R-squared = 39% to 50% (Table 6).

**Table 5:** Preliminary regression results for hedonic price models for rice.

	(1)	(2)	(3)	(4)
	All Income	High Income	Middle Income	Low Income
Percent broken	–0.0695***(0.0140)	–0.0450**(0.0208)	–0.0582*(0.0331)	–0.0031(0.0419)
GC	0.0394*(0.0223)	0.0595(0.0452)	–0.0238(0.0348)	0.0033(0.0430)
AAC	0.0954(0.0656)	0.2875(0.1944)	0.0569(0.1037)	0.0431(0.0932)
GT	0.2191(0.2245)	0.1964(0.3593)	0.9785*(0.5190)	–0.5421(0.5689)
Small Chalkiness	0.0121(0.0178)	0.1419**(0.0515)	–0.0291(0.0360)	–0.0061(0.0208)
Per capita income	0.0159**(0.0063)	0.0458(0.0308)	–0.0058(0.0365)	0.0118(0.0132)
Per capita rice consumption	0.0275**(0.0116)	0.0507**(0.0234)	–0.0083(0.0187)	0.0518**(0.0205)
Household size	0.0202(0.0130)	–0.0163(0.0340)	–0.0045(0.0416)	0.0425*(0.0237)
Age of rice purchaser	0.0338**(0.0151)	–0.0475(0.0416)	0.0405*(0.0238)	0.0066(0.0210)
Educ. of rice purchaser	0.0098(0.0137)	–0.0892*(0.0482)	0.0446**(0.0204)	–0.0095(0.0152)
Gender of rice purchaser	–0.0015(0.0124)	0.0132(0.0265)	0.0114(0.0174)	–0.0189(0.0207)
Location	–0.0216**(0.0109)	0.0319(0.0238)	–0.0256(0.0177)	–0.0497***(0.0173)
Intercept	1.9591**(0.9331)	1.5375(1.7302)	–0.7861(1.9570)	5.3386**(2.5060)
Observations	127	40	46	41
R-squared	0.39	0.70	0.47	0.42

Note: Standard errors in parentheses '***', '**', '*' significant at 1, 5, and 10%.

**Table 6:** Hedonic price function interacting income classes.

		Interaction with	
	Coefficients	Middle Income	Low Income
Percent broken	–0.0560***(0.0185)	–0.0078 (0.0398)	0.0395 (0.0538)
GC	0.0776**(0.0354)	–0.1055** (0.0508)	–0.0760 (0.0601)
AAC	0.1585(0.1583)	–0.1315 (0.1917)	–0.1447 (0.1925)
GT	–0.1008(0.3051)	1.0539* (0.5894)	–0.6394 (0.7458)
Small Chalkiness	0.0631(0.0392)	–0.0858 (0.0541)	–0.0637 (0.0456)
Per capita rice consumption	0.0592***(0.0191)	–0.0664** (0.0259)	–0.0449 (0.0290)
Middle income dummy	–3.3724(2.4746)		
Low income dummy	3.5979(3.2838)		
Household size	–0.0035(0.0132)		
Age of rice purchaser	0.0230(0.0160)		
Education of rice purchaser	0.0084(0.0132)		
Gender of rice purchaser	0.0029(0.0127)		
Location	–0.0225*(0.0114)		
Intercept	2.9427**(1.4672)		
Observations	127		
R-squared	0.50		

Note: Standard errors in parentheses '***', '**', '*' significant at 1, 5, and 10%.

The main advantages of the model presented in Table 6 are that this model maintains the original sample size and still shows the income effect on the revealed preferences of consumers. Results of this model suggest that only the high-income class significantly discounts broken grains. However, this is likely the result of high-income consumers purchasing rice from a wider range of quality classes, which is confirmed by the higher variability in the amount of percent broken in the rice samples obtained from this income class (Fig 1). Middle- and low-income consumers in this study did not purchase premium rice with low amounts of percent broken. Results also indicate that soft rice is preferred by high-income consumers with GC significant at five percent. Conversely, middle-income consumers discount rice with higher GC. This study also revealed that per capita rice consumption significantly affected WTP. However, the sign changes between high- and middle-income classes which indicate that high-income consumers spend more per kg of rice as their consumption increases and middle-income consumers spend less per kg of rice as their consumption increases. The results of high-income consumers’ preference for soft rice, as measured by GC values, is in agreement with previous studies [86].

### Implicit price

Marginal implicit prices are calculated as the product of the mean rice price from the collected samples and the mean beta coefficients of the physical and chemical characteristics divided by the mean of the explanatory variables of the collected rice samples. These implicit prices were estimated based on Table 5 for the whole sample as well as by income classes.

The data show that amount of percent broken grains was significant in almost all income classes. Although the magnitude of the implicit price is rather small for percent broken, it is important to note that there is a large range in percent broken values. Samples in this study ranged from 2.4% broken to 66.4% broken; the level of percent broken can change the price of rice by more than 0.08 USD (9.7%) throughout the total range. Gel consistency was found to be significant at the 10% level for the non-interacted variable with an implicit price of approximately 0.08 USD/kg for every 10% change and a total range of 550%. Also significant at the 10% level was GT for middle-income consumers. The implicit price for these consumers was 0.01 USD/kg but the range of values for GT in the middle-income was only 7.43°C. As such, GT could affect the price of rice by as much as 0.08 USD/kg (9.4%).

## Conclusion

This study was conducted to measure the contribution of grain quality attributes to the market value of rice in two rural towns in the Philippines. Unlike previous hedonic studies which considered semi-quantitative scores for measuring of GT and chalkiness, this study included measurement of GT based on thermal transitions in DSC and of chalkiness based on computerized image analyses. These techniques are more accurate and reliable than routinely used assays (alkali spreading test for GT and visual scores for chalkiness). Moreover, in addition to commonly used physicochemical data, this study also employed an interaction term for income classes to reveal income effects in these factors on rice price.

The results of this study indicate that consumers’ response to grain quality characteristics changes over income classes. Generally speaking, low-income consumers appear to have less pronounced preferences for rice based on physical and chemical characteristics. Or more likely, these consumers do not have the economic power to express their preferences. Additionally, the absence of preference may result from homogeneity in the rice consumed by this income class. High-income consumers have the largest variability in rice grain quality attributes and concurrently appear to have the most pronounced preferences among consumers. High-income consumers also spend more money per kg as their consumption increases, while the opposite has been observed for the middle-income class.

These results provide important insights into value chain upgrading as the Philippines is currently struggling to reduce imports, increase rice self-sufficiency and raise income of poor farmers. Greater head rice recovery, for example, is consistently emphasized as a priority trait by consumers in the Philippines since the 1980s [44,47,58]. Our hedonic analysis revealed that greater percentage of broken rice grains (i.e. lower head rice recovery rate) is still discounted by consumers in medium and high income classes today. Therefore, in order to enable Filipino farmers to access those market segments with local rice, more investment will be needed in upgrading of pre- and post-milling operations (separation of varieties, sorting, and grading) as well as through rice breeding [87]. More generally, our findings can be used by rice breeders for setting priorities and incorporating grain quality improvements in varietal development, along with agronomic and stress-tolerance traits. Issues of grain quality and postharvest losses are likely to become more pronounced in the future as heat stress can reduce milled rice yields by as much as 13.8% for every 1°C increase in the average growing season temperature [88] and annual mean temperatures in all areas of the Philippines are expected to increase by as much as 1.1°C by 2020 and 2.2°C by 2050 compared to baseline temperatures from 1971–2000 [89].

## Supporting Information

S1 Fig Distribution of chalkiness, grain length, grain width, percentage of head rice from the milled grain, and length-width ratio of the grain length of raw milled rice grains submitted by respondents from Famy and Sta Maria. (TIFF)

S2 Fig Distribution of AAC, GT, and GC of the rice samples obtained from the respondents in Famy and Sta Maria. (TIFF)

## References

[1] Khush GS. What it will take to feed 5.0 billion rice consumers in 2030. Plant molecular biology. 2005;59: 1–6. 10.1007/s11103-005-2159-5 16217597

[2] Calpe C. Status of the world rice market in 2002 20th Session of the International Rice Commission. Bangkok, Thailand: Food and Agriculture Organization of the United Nations; 2003.

[3] Deaton AS, Dreze J. Nutrition in India: Facts and Interpretations. SSRN Electronic Journal. 2008; 42–65. 10.2139/ssrn.1135253

[4] Demont M, Zossou E, Rutsaert P, Ndour M, Van Mele P, Verbeke W. Consumer valuation of improved rice parboiling technologies in Benin. Food Quality and Preference. Elsevier Ltd; 2012;23: 63–70. 10.1016/j.foodqual.2011.07.005

[5] Jensen RT, Miller NH. Giffen Behavior and Subsistence Consumption. The American economic review. 2008;98: 1553–1577. 10.1257/aer.98.4.1553 21031158 PMC2964162

[6] Shah CH. Food Preference, Poverty, and the Nutrition Gap. Economic Development and Cultural Change. 1983;32: 121 10.1086/451374

[7] Rutsaert P, Demont M, Verbeke W. Consumer Preferences for Rice in Africa In: Wopereis MCS, Johnson D, Ahmad N, Tollens E, Jalloh A, editors. Realizing Africa’s Rice Promise. Boston: CABI Publishing; 2013 pp. 293–301.

[8] Minten B, Murshid KAS, Reardon T. Food Quality Changes and Implications: Evidence from the Rice Value Chain of Bangladesh. World Development. 2013;42: 100–113. 10.1016/j.worlddev.2012.06.015

[9] Adair CR, Beachell HM, Jodon NE, Johnston TH, Thysell JR, Green VE Jr, et al Rice Breeding and Testing Methods in the United States Rice in the United States: Varieties and Production. Washington DC: United States Department of Agriculture; 1966 pp. 19–64.

[10] Graham R. A Proposal for IRRI to Establish a Grain Quality and Nutrition Research Center. Los Baños, Philippines; 2002. Report No.: 44.

[11] Tomlins K, Manful J, Gayin J, Kudjawu B, Tamakloe I. Study of sensory evaluation, consumer acceptability, affordability and market price of rice. Journal of the Science of Food and Agriculture. 2007;87: 1564–1575. 10.1002/jsfa.2889

[12] Codex Alimentarius Commission. Codex Standard for Rice. 1995.

[13] Council of the European Eunion. Council Regulation (EC) 1785/2003 on the common organisation of the market in rice. Official Journal of the European Union. 2003;270: 96–113.

[14] Dela Cruz NM, Khush GS. Rice Grain Quality Evaluation Procedures In: Singh RK, Singh US, Khush GS, editors. Aromatic Rices. New Delhi, India: Mohan Primlani; 2000 pp. 15–28.

[15] Fitzgerald MA, Resurreccion AP. Maintaining the yield of edible rice in a warming world. Functional Plant Biology. 2009;36: 1037 10.1071/FP09055 32688715

[16] Ikehashi H, Khush GS. Methodology of assessing appearance of the rice grain, including chalkiness and whiteness Workshop on Chemical Aspects of Rice Grain Quality. Los Baños, Philippines: International Rice Research Institute; 1979 pp. 223–230.

[17] Yoshioka Y, Iwata H, Tabata M, Ninomiya S, Ohsawa R. Chalkiness in Rice: Potential for Evaluation with Image Analysis. Crop Science. 2007;47: 2113 10.2135/cropsci2006.10.0631sc

[18] Juliano BO, Perez CM, Barber S, Blakeney AB, Iwasaki TA, Shibuya N, et al International Cooperative Comparison of instrument methods for cooked rice texture. Journal of Texture Studies. 1981;12: 17–38. 10.1111/j.1745-4603.1981.tb00533.x

[19] Juliano BO. A Simplified Assay for Milled-Rice Amylose. Cereal Science Today. 1971;16: 334–340.

[20] Mcgrance SJ, Cornell HJ, Rix CJ. A Simple and Rapid Colorimetric Method for the Determination of Amylose in Starch Products. Starch—Stärke. 1998;50: 158–163.

[21] Perez CM, Juliano BO. Modification of the Simplified Amylose Test for Milled Rice. Starch—Stärke. 1978;30: 424–426. 10.1002/star.19780301206

[22] Delwiche SR, Mckenzie KS, Webb BD. Quality Characteristics in Rice by Near-Infrared Reflectance Analysis of Whole-Grain Milled Samples. Cereal Chemistry. 1996;73: 257–263.

[23] Fitzgerald MA, Bergman CJ, Resurreccion AP, Möller J, Jimenez R, Reinke RF, et al Addressing the Dilemmas of Measuring Amylose in Rice. Cereal Chemistry. 2009;86: 492–498. 10.1094/CCHEM-86-5-0492

[24] Morrison WR, Azudin MN. Variation in the amylose and lipid contents and some physical properties of rice starches. Journal of Cereal Science. 1987;5: 35–44. 10.1016/S0733-5210(87)80007-3

[25] Vilaplana F, Hasjim J, Gilbert RG. Amylose content in starches: Toward optimal definition and validating experimental methods. Carbohydrate Polymers. 2012;88: 103–111. 10.1016/j.carbpol.2011.11.072

[26] Kumar I, Khush GS. Genetic Analysis of Different Amylose Levels in Rice. Crop Science. 1987;27: 1167 10.2135/cropsci1987.0011183X002700060016x

[27] Calingacion M, Laborte A, Nelson A, Resurreccion A, Concepcion JC, Daygon VD, et al Diversity of global rice markets and the science required for consumer-targeted rice breeding. PloS one. 2014;9: e85106 10.1371/journal.pone.0085106 24454799 PMC3893639

[28] Cagampang GB, Perez CM, Juliano BO. A gel consistency test for eating quality of rice. Journal of the Science of Food and Agriculture. 1973;24: 1589–1594. 10.1002/jsfa.2740241214 4771843

[29] Antonio AA, Juliano BO. Physiochemical properties of glutinous rices in relation to pinipig. Philippine Agricultural Scientist. 1974;58: 17–23.

[30] Cuevas RP, Daygon VD, Corpuz HM, Nora L, Reinke RF, Waters DLE, et al Melting the secrets of gelatinisation temperature in rice. Functional Plant Biology. 2010;37: 439 10.1071/FP09258

[31] Singh N, Sodhi NS, Kaur M, Saxena SK. Physico-chemical, morphological, thermal, cooking and textural properties of chalky and translucent rice kernels. Food Chemistry. 2003;82: 433–439. 10.1016/S0308-8146(03)00007-4

[32] Cuevas RP, Fitzgerald MA. Genetic Diversity of Rice Grain Quality In: Caliskan M, editor. Genetic Diversity in Plants. InTech; 2012 pp. 285–310. Available: http://www.intechopen.com/books/genetic-diversity-in-plants

[33] Little RR, Hilder GB, Dawson EH. Differential effect of dilute alkali on 25 varieties of milled white rice. Cereal Chemistry. 1958;35: 111–126.

[34] Juliano BO. Asian perspective on rice sensory quality. Cereal Foods World. 2001;46: 531–535.

[35] Kaosa-ard M, Juliano BO. Assessing rice quality characteristics and prices in selected international markets In: Juliano BO, Pollard LR, Argosino G, editors. Rice Grain Marketing and Quality Issues. Los Baños, Philippines: International Rice Research Institute; 1991 pp. 23–36.

[36] Spiller K. It tastes better because … consumer understandings of UK farmers’ market food. Appetite. 2012;59: 100–7. 10.1016/j.appet.2012.04.007 22521516

[37] Brorsen BW, Grant WR, Rister ME. A Hedonic Price Model for Rough Rice Bid/Acceptance Markets. American Journal of Agricultural Economics. 1984;66: 156 10.2307/1241032

[38] Combris P, Lecocq S, Visser M. Estimation of a Hedonic Price Equation for Bordeaux Wine: Does Quality Matter? The Economic Journal. 1997;107: 390–402.

[39] Nerlove M. Hedonic price functions and the measurement of preferences: The case of Swedish wine consumers. European Economic Review. 1995;39: 1697–1716. 10.1016/0014-2921(95)00013-5

[40] Deodhar SY, Intodia V. What’s in a Drink You Call a Chai? Quality Attributes and Hedonic Price Analysis of Tea. Journal of International Food and Agribusiness Marketing. 2004;16: 43–57.

[41] Carew R, Smith EG. The value of apple characteristics to wholesalers in western Canada : A hedonic approach. Canadian Journal of Plant Science. 2004;84: 764–771.

[42] Stanley LR, Tschirhart J. Hedonic Prices for a Nondurable Good: The Case of Breakfast Cereals. The Review of Economics and Statistics. 1991;73: 537 10.2307/2109582

[43] Hulten CR. Price Hedonics: A Critical Review. FBNY Economic Policy Review. 2003;9: 5–15.

[44] Abansi CL, Duff B, Lantican FA, Juliano BO. Consumer demand for rice grain quality in selected rural and urban markets in the Philippines In: Unnevehr L, Duff B, Juliano B, editors. Consumer Demand for Rice Grain Quality. Los Baños, Philippines: International Rice Research Institute; 1992.

[45] Wang Z, Zheng S, Lambert DM, Fukuda S. A hedonic price model for rice market in China. Journal of the Faculty of Agriculture Kyushu University. 2009;54: 541–548.

[46] Dalton T. A household hedonic model of rice traits: economic values from farmers in West Africa. Agricultural Economics. 2004;31: 149–159.

[47] Unnevehr LJ. Consumer Demand for Rice Grain Quality and Returns to Research for Quality Improvement in Southeast Asia. American Journal of Agricultural Economics. 1986;68: 634–641.

[48] Laborte AG, Paguirigan NC, Moya PF, Nelson A, Sparks AH, Gregorio GB. Farmers’ Preference for Rice Traits: Insights from Farm Surveys in Central Luzon, Philippines, 1966–2012. Plos One. 2015;10: e0136562 10.1371/journal.pone.0136562 26317505 PMC4552743

[49] Bautista RC, Siebenmorgen TJ, Counce PA. Rice Kernel Chalkiness and Milling Quality Relationship of Selected Cultivars In: Norman RJ, Moldenhauer KA, editors. BR Wells Rice Research Studies 2009. Fayetteville, Arkansas: University of Arkansas Division of Agriculture; 2010 pp. 220–229.

[50] Chen Y-R, Chao K, Kim MS. Machine vision technology for agricultural applications. Computers and Electronics in Agriculture. 2002;36: 173–191. 10.1016/S0168-1699(02)00100-X

[51] Reuss R. Using real-time quality measurement to maintain and increase value across the grain supply chain In: Wright EJ, Webb MC, Highley E, editors. Stored Grains in Australia. Canberra, Australia: CSIRO Stored Grain Research Laboratory; 2003.

[52] Normand FL, Marshall WE. Differential Scanning Calorimetry of Whole Grain Milled Rice and Milled Rice Flour. Cereal Chemistry. 1989;66: 317–320.

[53] Slade L, Levine H. Non-equilibrium melting of native granular starch: Part I. Temperature location of the glass transition associated with gelatinization of A-type cereal starches. Carbohydrate Polymers. 1988;8: 183–208. 10.1016/0144-8617(88)90002-1

[54] Bason ML, Ronalds JA, Wrigley CW, Hubbard LJ. Testing for sprout damage in malting barley using the Rapid Visco-Analyzer. Cereal Chemistry. 1993;70: 269–272.

[55] Champagne ET, Bett KL, Vinyard BT, McClung AM, Barton FE, Moldenhauer K, et al Correlation Between Cooked Rice Texture and Rapid Visco Analyser Measurements. Cereal Chemistry. 1999;76: 764–771. 10.1094/CCHEM.1999.76.5.764

[56] Chen M-H, Bergman C, Pinson S, Fjellstrom R. Waxy gene haplotypes: Associations with apparent amylose content and the effect by the environment in an international rice germplasm collection. Journal of Cereal Science. 2008;47: 536–545. 10.1016/j.jcs.2007.06.013

[57] Juliano BO, Onate LU, del Mundo AM. Relation of starch composition, protein content, and gelatinization temperature to cooking and eating qualities of milled rice. Food Technology. 1965;19: 116–121.

[58] Juliano BO, Villareal CP. Grain Quality Evaluation of World Rices. Los Baños, Philippines: International Rice Research Institute; 1993.

[59] NSO. 2012 FAMILY INCOME AND EXPENDITURE SURVEY Final Results: National Capital Region. 2014.

[60] International Organization for Standardization. ISO 6647–2: 2007–Rice—Determination of amylose content—Part 2: Routine methods. 10. 2007.

[61] International Organization for Standardization. ISO 6647–1: 2007–Rice—Determination of amylose content—Part 1: Reference method. 11. 2007.

[62] American Association of Ceral Chemists. Approved methods of the American Association of Cereal Chemists (10th ed.). St. Paul, Minnesota; 2000.

[63] Fitzgerald MA, Martin M, Ward RM, Park WD, Shead HJ. Viscosity of rice flour: a rheological and biological study. Journal of agricultural and food chemistry. 2003;51: 2295–9. 10.1021/jf020574i 12670173

[64] Lancaster KJ. A New Approach to Consumer Theory. Journal of Political Economy. 1966;74: 132–157.

[65] Lusk JL, Shogren JF. Experimental Auctions. Cambridge, MA: Cambridge University Press; 2008.

[66] Suwannaporn P, Linnemann A. Consumer Preferences and Buying Criteria in Rice: A Study to Identify Market Strategy for Thailand Jasmine Rice Export. Journal of Food Products Marketing. 2008;14: 33–53. 10.1080/10454440801986348

[67] Champagne ET, Bett-Garber KL, Fitzgerald MA, Grimm CC, Lea J, Ohtsubo K, et al Important Sensory Properties Differentiating Premium Rice Varieties. Rice. 2010;3: 270–281. 10.1007/s12284-010-9057-4

[68] Del Mundo AM, Juliano BO. Consumer Preference and Properties of Raw and Cooked Milled Rice. Journal of Texture Studies. 1981;12: 107–120. 10.1111/j.1745-4603.1981.tb01225.x

[69] Abansi CL, Lantican FA, Duff B, Juliano BO. Hedonic Model Estimation: Application to Consumer Demand for Rice Grain Quality. Transactions of the National Academy of Science and Technology. 1990;12: 235–256.

[70] Lantican FA, Quilloy KP, Sombilla MA. Estimating the Demand Elasticities of Rice in the Philippines. Los Baños, Philippines; 2011.

[71] Juliano BO, Perez CM, Maranan CL, Abansi CL, Duff B. Grain quality characteristics of rice in Philippine retail markets In: Unnevehr LJ, Duff B, Juliano BO, editors. Consumer Demand for Rice Grain Quality. Los Baños, Philippines: International Rice Research Institute; 1992 pp. 77–86.

[72] Roferos LT, Felix A dR, Juliano BO. The Search for the Grain Quality of Raw and Cooked IR64 Milled Rice Among Philippine Seed Board Rice Varieties. The Philippine Agricultural Scientist. 2006;89: 58–70.

[73] Tashiro T, Wardlaw I. The effect of high temperature on kernel dimensions and the type and occurrence of kernel damage in rice. Australian Journal of Agricultural Research. 1991;42: 485–496. 10.1071/AR9910485

[74] Ashida K, Iida S, Yasui T. Morphological, Physical, and Chemical Properties of Grain and Flour from Chalky Rice Mutants. Cereal Chemistry. 2009;86: 225–231. 10.1094/CCHEM-86-2-0225

[75] Zhao X, Fitzgerald MA. Climate change: implications for the yield of edible rice. PloS one. 2013;8: e66218 10.1371/journal.pone.0066218 23776635 PMC3680399

[76] Semple RL, Hicks PA, Lozare JV, Castermans A. Towards integrated commodity and pest management in grain storage Regional Training Course on Integrated Pest Management Strategies in Grain Storage Systems. Rome, Italy: Food and Agriculture Organization of the United Nations; 1992 p. 526.

[77] Rickman JF. Grain quality from harvest to market 9th JIRCAS International Symposium–“Value-Addition to Agricultural Products”. Tsukuba, Japan: Japan International Research Center for Agricultural Sciences; 2002 pp. 94–98.

[78] Rachmat R, Thahir R, Gummert M. The Emperical Relationship Between Price and Quality of Rice at Market Level in West Java. Indonesian Journal of Agricultural Science. 2006;7: 27–33.

[79] Hamaker BR, Griffin VK. Effect of disulfide bond-containing protein on rice starch gelatinization and pasting. Cereal Chemistry. 1993;70: 377–380.

[80] Liang X, King JM, Shih FF. Pasting Property Differences of Commercial and Isolated Rice Starch with Added Lipids and β-Cyclodextrin. Cereal Chemistry. 2002;79: 812–818. 10.1094/CCHEM.2002.79.6.812

[81] Maniñgat CC, Juliano BO. Starch Lipids and Their Effect on Rice Starch Properties. Starch—Stärke. 1980;32: 76–82. 10.1002/star.19800320303

[82] Martin M, Fitzgerald MA. Proteins in Rice Grains Influence Cooking Properties! Journal of Cereal Science. 2002;36: 285–294. 10.1006/jcrs.2001.0465

[83] Jane J, Chen YY, Lee LF, McPherson AE, Wong KS, Radosavljevic M, et al Effects of Amylopectin Branch Chain Length and Amylose Content on the Gelatinization and Pasting Properties of Starch 1. Cereal Chemistry. 1999;76: 629–637. 10.1094/CCHEM.1999.76.5.629

[84] Nakamura Y, Sakurai A, Inaba Y, Kimura K, Iwasawa N, Nagamine T. The fine Structure of Amylopectin in Endosperm from Asian Cultivated Rice can be largely Classified into two Classes. Starch—Stärke. 2002;54: 117–131. 10.1002/1521-379X(200204)54:3/4<117::AID-STAR117>3.0.CO;2-2

[85] Juliano BO, Perez CM, Resurreccion AP. Apparent Amylose Content and Gelatinization Temperature Types of Philippine Rice Accessions in the IRRI Gene Bank. Philippine Agricultural Scientist. 2009;92: 106–109.

[86] Bartell KH. Fine Filipino Food All Editions of Fine Filipino Food. New York: Hippocrene Books; 2003.

[87] IRRI. Rice Knowledge Bank. In: Breeding for grain quality [Internet]. 2006 Accessed: 12 October 2015. Available: http://www.knowledgebank.irri.org/ricebreedingcourse/Grain_quality.htm

[88] Lyman NB, Jagadish KS V, Nalley LL, Dixon BL, Siebenmorgen T. Neglecting rice milling yield and quality underestimates economic losses from high-temperature stress. PloS one. 2013;8: e72157 10.1371/journal.pone.0072157 23991056 PMC3750041

[89] PAGASA. Climate change scenarios in the Philippines. Quezon City; 2011.

