# Supplementary figures and images for: Rice Grain Quality and Consumer Preferences: A Case Study of Two Rural Towns in the Philippines
Source: PLoS One. 2016 Mar 16;11(3):e0150345. doi: 10.1371/journal.pone.0150345 (PMC4794204; doi:10.1371/journal.pone.0150345)

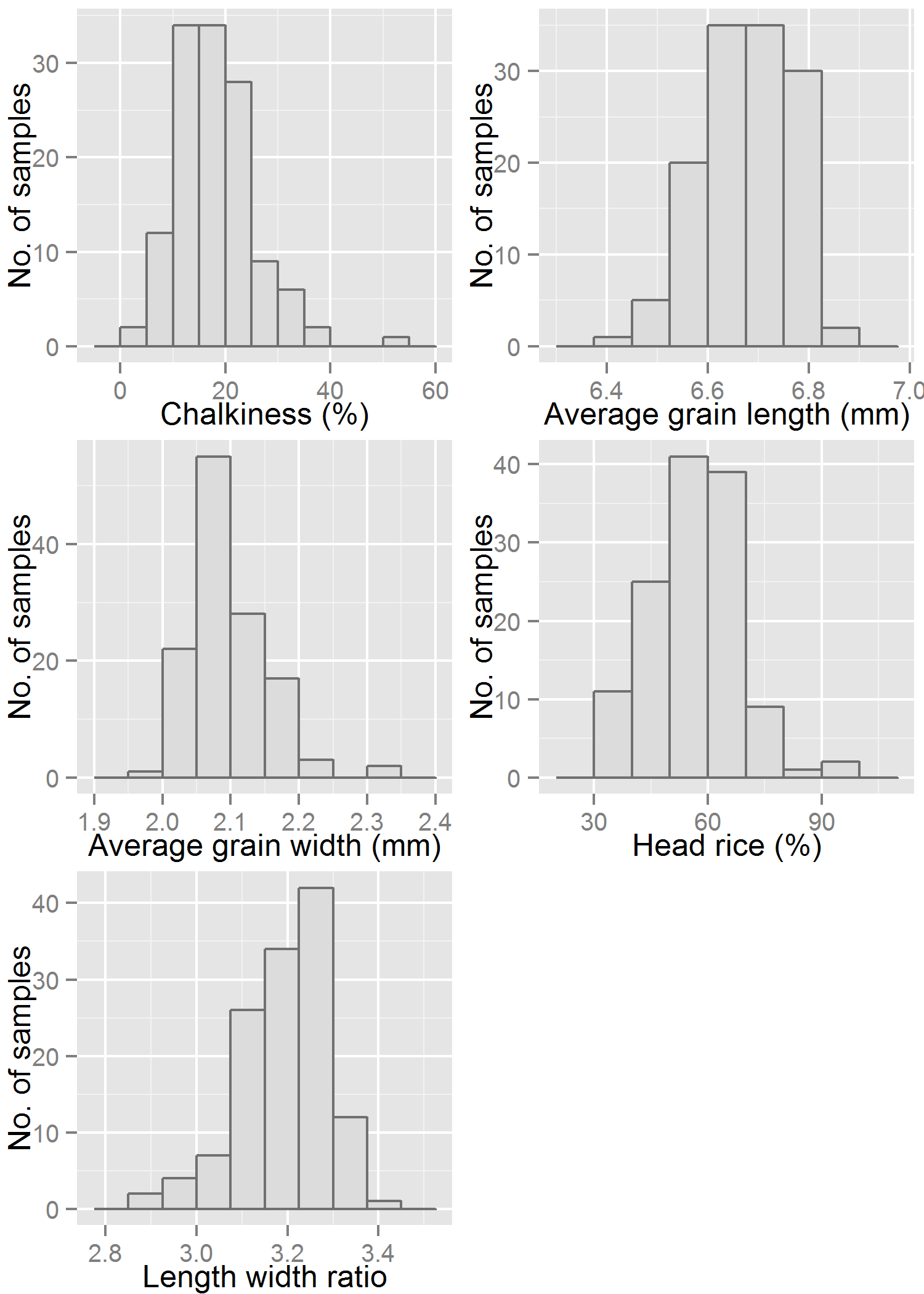

Supplement: S1 Fig — (TIFF) [file pone.0150345.s001.tiff]

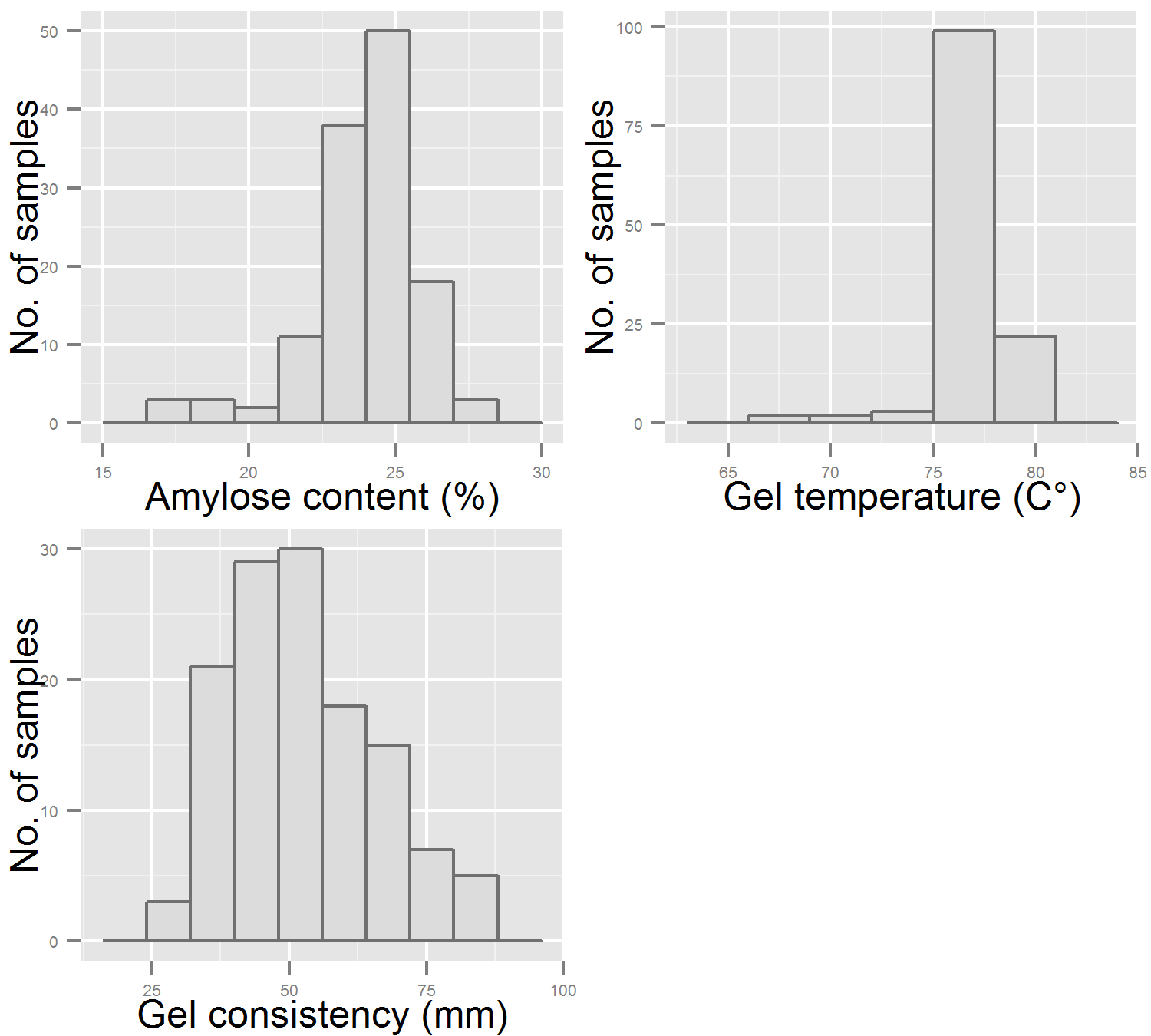

Supplement: S2 Fig — (TIFF) [file pone.0150345.s002.tiff]
